# Neutrophil Dysfunction in the Airways of Children with Acute Respiratory Failure Due to Lower Respiratory Tract Viral and Bacterial Coinfections

**DOI:** 10.1038/s41598-019-39726-w

**Published:** 2019-02-27

**Authors:** Jocelyn R. Grunwell, Vincent D. Giacalone, Susan Stephenson, Camilla Margaroli, Brian S. Dobosh, Milton R. Brown, Anne M. Fitzpatrick, Rabindra Tirouvanziam

**Affiliations:** 1 Emory University School of Medicine, Department of Pediatrics, Atlanta, GA USA; 2 Children’s Healthcare of Atlanta at Egleston, Atlanta, GA USA

## Abstract

Neutrophils are recruited to the airways of patients with acute respiratory distress syndrome (ARDS) where they acquire an activated pro-survival phenotype with an enhanced respiratory burst thought to contribute to ARDS pathophysiology. Our *in vitro* model enables blood neutrophil transepithelial migration into cell-free tracheal aspirate fluid from patients to recapitulate the primary airway neutrophil phenotype observed *in vivo*. Neutrophils transmigrated through our model toward airway fluid from children with lower respiratory viral infections coinfected with bacteria had elevated levels of neutrophil activation markers but paradoxically exhibited an inability to kill bacteria and a defective respiratory burst compared with children without bacterial coinfection. The airway fluid from children with bacterial coinfections had higher levels of neutrophil elastase activity, as well as myeloperoxidase levels compared to children without bacterial coinfection. Neutrophils transmigrated into the aspirate fluid from children with bacterial coinfection showed decreased respiratory burst and killing activity against *H*. *influenzae* and *S*. *aureus* compared to those transmigrated into the aspirate fluid from children without bacterial coinfection. Use of a novel transmigration model recapitulates this pathological phenotype *in vitro* that would otherwise be impossible in a patient, opening avenues for future mechanistic and therapeutic research.

## Introduction

Viral lower airway infections are common in infants and preschool children, and the severity respiratory symptoms can vary from mild cough and congestion to respiratory failure requiring endotracheal intubation and mechanical ventilation. We cannot predict which children will recover quickly versus those who will progress to severe ARDS. No ARDS-specific pharmacological treatments exist, rather treatment is supportive using lung-protective ventilation strategies and the administration of antibiotics for suspected bacterial infection. The study of immune regulation in children is needed because both the lung and immunity are maturing during this critical stage of development and the primary cause of PARDS is direct lung injury due to infection^1,2^.

ARDS is characterized by refractory hypoxemia and non-cardiogenic pulmonary edema due to damage to the lung epithelium and pulmonary endothelium. In adults with ARDS increased counts of airway neutrophils are associated with more severe lung injury and mortality^3,4^. Airway neutrophils from adults with ARDS have enhanced survival, a primed respiratory burst response, and increased phagocytic capacity^5^. In patients with pulmonary diseases characterized by chronic bacterial infections, such as cystic fibrosis, airway neutrophils inhibit T-cell function via two pathways — by depletion of arginine following degranulation and activation of Arginase-1 (Arg1), and by activation of the programmed death ligand-1 (PD-L1)/programmed death-1 (PD-1) axis, resulting in reduced proliferation and impaired effector functions^6–15^. By contrast, pulmonary environmental influence on immune cell regulation is poorly characterized in PARDS due to the lack of a model system able to recapitulate the airway neutrophil phenotype in severe paediatric lung injury.

Clinical information obtainable at the bedside is often used to prognosticate yet is insufficient for determining the pathobiology of the lung injury. Understanding the biology of neutrophils recruited to the lung during PARDS is crucial for advancing prognostication, risk stratification, and development of novel therapeutic strategies for children who progress to severe PARDS. Herein we hypothesized that markers of degranulation on the surface of airway neutrophils and in the cell-free airway fluid within 24-hours of intubation would be associated with bacterial respiratory co-infection. We used an *in vitro* model based on blood neutrophil transepithelial migration into the airway fluid of endotracheally intubated children with suspected or confirmed lower airway viral infections at risk for progressing to or with PARDS^16^. We hypothesized that neutrophils recruited to the cell-free airway fluid of children with bacterial coinfections would have a defective respiratory burst and ability to kill bacteria compared to children with no bacterial coinfection.

## Materials and Methods

### Human subjects

This prospective observational study was performed in the paediatric intensive care unit (PICU) at Children’s Healthcare of Atlanta at Egleston from January to April 2018. The study was approved by the Institutional Review Board at Emory University, and we confirm that all research was performed in accordance with relevant guidelines and regulations. Informed consent was obtained from the parents of all subjects prior to collection and use of their samples.

All patients greater than 48 hours of age, with a corrected gestational age of at least 40 weeks, who were 18 years old or younger admitted to the PICU, and who met criteria for being at risk or having PARDS as defined by the Pediatric Acute Lung Injury Consensus Conference (PALICC)^2^ were screened for eligibility. To be enrolled in the study, children had to have lung injury within 7 days of a known clinical insult, new infiltrate(s) consistent with acute pulmonary parenchymal disease on chest imaging and be receiving oxygen delivered either non-invasively or invasively to maintain an oxygen saturation in the 88–97% range. Respiratory viral infections were confirmed by respiratory viral polymerase chain reaction testing as ordered at the discretion of the primary medical team caring for the patient. Although a viral infection may have been suspected, not all patients had clinical identification of the virus by laboratory testing.

Children were excluded if they had any perinatal related lung disease, respiratory failure fully explained by cardiac failure or fluid overload, chronic respiratory failure with mechanical ventilation via a tracheostomy or RAM cannula, confirmed immunodeficiency disorder, immunosuppression from chemotherapy for an oncologic process, chronic immunosuppression in a bone marrow transplant or solid organ transplant recipient, no parent or legal guardian present to provide written informed consent, or the attending physician did not wish the patient to participate in the study.

### Clinical data collection

Clinical data were abstracted from the medical record onto a standardized form. Variables included demographics; fraction inspired oxygen, mean airway pressure, arterial oxygen saturation or arterial oxygen pressure used to calculate an oxygen saturation index (OSI) or oxygenation index (OI), respectively; laboratory and microbiology results; length of mechanical ventilation and need for reintubation; length of PICU stay, use of high frequency oscillatory ventilation (HFOV) or extracorporeal membrane oxygenation (ECMO); and vital status. Severity of illness was determined by the Pediatric Risk of Mortality (PRISM)-III and Pediatric Logistic Organ Dysfunction (PELOD) scores were calculated within 24 hours of PICU admission^17–20^. Need for mechanical ventilation to 28-days was monitored to calculate ventilator-free days^21^. Lung injury severity was categorized according to PALICC criteria^2^ as follows: At Risk = OI < 4 (OSI < 5); Mild = 4 ≤ OI < 8 (5 ≤ OSI < 7.5); Moderate = 8 ≤ OI < 16 (7.5 ≤ OSI < 12.3); Severe = OI ≥ 16 (OSI ≥ 12.3).

### Blood and tracheal aspirate sample collections

A 4 mL blood specimen was collected in a K-EDTA vacutainer (Becton Dickinson, Franklin Lakes, NJ) within 24 hours of endotracheal intubation for children weighing greater than 6 kg who had either an arterial or central venous catheter. Serial blood samples were collected every other day for up to one week, and then weekly thereafter, for the duration of mechanical ventilation and/or the presence of either an arterial or central venous catheter. Assays for neutrophil activation were performed within an hour of blood collection and cells were kept at 4 °C until the assay was performed.

Tracheal aspirates were obtained from patients on conventional mechanical ventilation by instilling 1–5 mL of sterile saline through the inline Ballard suction catheter and into a sterile Luken’s trap as part of routine suctioning. Serial tracheal aspirate samples were collected every other day for up to one week, and then weekly thereafter, for the duration of mechanical ventilation. There was no weight limit below which a tracheal aspirate could be obtained on a patient. Children who were mechanically ventilated with high frequency oscillation ventilation were suctioned only if clinically indicated and approved by the attending physician. Tracheal aspirate samples were immediately placed on ice for transport to the laboratory for processing.

### Blood and tracheal aspirate sample processing

Blood was spun at 400 × g to separate cells from platelet-rich plasma. Plasma was spun at 3,000 × g to generate platelet-free plasma, aliquoted, and stored at −80 °C. Pelleted blood cells were resuspended in phosphate buffered saline (PBS) with 2.5 mM ethylenediaminetetraacetic acid (EDTA) up to the original whole blood volume for flow cytometry analysis or for neutrophil purification, as described^22^. Neutrophils were purified by negative selection from PBS-EDTA-washed whole blood using the EasySep Direct Human Neutrophil Isolation kit (STEMCELL Technologies Inc., Cambridge, MA) according to the manufacturer’s instructions.

Tracheal aspirate was gently dissociated using repeated passage through an 18 G needle after the addition of 6 ml of PBS-EDTA. Dissociated tracheal aspirate was then centrifuged at 800 × g to generate a cell pellet and a fluid fraction. The fluid fraction was spun at 3,000 × g to generate cell-free airway supernatant (ASN), aliquoted, and stored at −80 °C. Airway cells were resuspended in PBS-EDTA, counted using a Countess cytometer, and used for flow cytometry assays.

### Transepithelial migration model for neutrophil conditioning

We used an *in vitro* model recently developed by our group that recapitulates airway neutrophil phenotype in chosen pathological lung environments. This model was previously applied to cystic fibrosis (CF), asthma, and chronic obstructive pulmonary disease (COPD), using ASN from patients with these conditions added apically to attract and condition naive blood neutrophils through a small airway epithelial lining into the lumen^16^. Briefly, 2 × 10^6^ purified blood neutrophils were loaded onto the 200 µm-thick basal compartment of an Alvetex scaffold (ReproCELL, Glasgow, UK) coated with collagen and a monolayer of H441 cells, a human Club cell line, grown for two weeks at an air-liquid interface^16^. Neutrophils were allowed to migrate for 14 hours at 37 °C at 5% CO_2_ through the collagen and epithelial layers into the apical compartment comprised of ASN diluted to 33% in RPMI 1640 medium with L-glutamine, or the same medium with the chemoattractant leukotriene B4 (LTB4, 100 nM).

### Cell staining and flow cytometry

Blood and airway neutrophils collected from patients *in vivo* and neutrophils transmigrated in the *in vitro* model were preincubated with human TruStain FcX receptor blocking solution (BioLegend, San Diego, CA) and Live/Dead Aqua (Thermo Fisher Scientific, Waltham, MA) for 10 min on ice in the dark followed by labeling antibodies listed in Table E1, for 30 min. All panels were applied to blood samples. Due to limited numbers of cells in some tracheal aspirate samples, flow cytometry panels (detailed in Table E1) were prioritized as follows: Panel 1, Airway Purity, Fluorescence Minus 5 Control, Panel 2, and Panel 3. Samples were treated with BD Phosflow™ Lyse/Fix Buffer (BD Biosciences, San Jose, CA), washed twice with PBS-EDTA, and stored at 4 °C in the dark until acquisition on a CytoFLEX flow cytometer (Beckman Coulter, Indianapolis, IN). Transmigrated neutrophils were similarly stained with flow panels described in Table E1 and acquired on an LSRII cytometer (BD Biosciences).

Compensation, using AbC Total Antibody and ArC Amine-Reactive Compensation Beads (Thermo Fisher Scientific), gating and analysis were performed with FlowJo v.10 (Tree Star, Ashland, OR). Single cells were separated from doublet cells by gating on forward scatter area versus forward scatter height. Neutrophils were selected based on their forward scatter area versus side scatter area profiles. Live cells were then selected by exclusion of dead events positive for the dye Live/Dead Aqua, and platelets or platelet/leukocyte aggregates were excluded by staining for CD41a^+^ events. CD66b^+^ neutrophils were then confirmed by gating on CD66b versus side scatter area for both tracheal aspirate (Fig. E1A) and whole blood neutrophils (Fig. E1B). Mean fluorescence intensities of surface markers are reported for CD66b^+^ neutrophils. To determine the background mean fluorescence intensity signal in the five reporter channels (PB450, AF488, PECy7, PE, and APC) we report mean fluorescence signal intensity in CD66b + neutrophils from the blood and airway samples that were only stained for CD41a, Live/Dead aqua, CD66b, and CD16 (fluorescence minus (FM) five) controls (Fig. E2).

### Human neutrophil elastase activity and enzyme-linked immunosorbent assays (ELISAs)

Human neutrophil elastase (HNE) activity was measured in ASN and plasma according to the supplier’s protocol (Cayman Chemical, Ann Arbor, MI), with a kit-supplied standard curve. In addition, human matrix metalloproteinase-9 (MMP-9) DuoSet, myeloperoxidase (MPO) Quantikine ELISA kits (R&D Systems, Minneapolis, MN), and human lactoferrin ELISA kit (Abcam, Cambridge, MA) were used according to suppliers’ protocols, and protein concentrations calculated using supplied standard curves. Data were collected on a BioTek (Winooski, VT) Synergy HT 96-well plate reader with recommended wavelengths and standard curve fit procedures.

### Neutrophil respiratory burst assay

Neutrophil respiratory burst intensity was assessed with dihydrorhodamine 123 after stimulation with 100 ng/mL of *N*-formyl-methionyl-leucyl-phenylalanine (fMLF) as described using a FACSCalibur cytometer^23,24^. Phorbol 12-myristate 13-acetate (PMA) was used at a final concentration of 200 nM as a positive control for activation.

### Bacterial killing assay

*S*. *aureus* and *H*. *influenzae* were grown in Luria broth (LB) or Chocolate media, respectively, at 37 °C with aeration to an optical density (600 nm) of 0.5. Bacteria were pelleted by centrifugation at 14,000 × *g* for 1 min at room temperature, resuspended in 500 µL 10% human serum in RPMI and opsonized for 30 min at 37 °C. Opsonized bacteria were added to neutrophils at a multiplicity of infection of 1. After 1 h of incubation at 37 °C, the cultures were lysed using 0.1% Triton for 2 min, after which the bacteria were serially diluted and plated in triplicate on LB or Chocolate agar plates, respectively, overnight at 37 °C in a non-CO_2_ incubator. Viable bacterial colony forming unit (CFUs) were counted the following day and reported as a percentage of bacterial counts recorded from bacteria incubated in absence of neutrophils.

### Data analysis

Statistics were performed using JMP Pro 13 (SAS Institute, Cary, NC) and Prism 7 (GraphPad, San Diego, CA) for Windows. Unless otherwise stated, comparisons between samples were made using a Mann-Whitney U test or ANOVA with *post-hoc* Tukey test for multiple comparisons. Statistical significance was defined as a *p* value less than 0.05.

## Results

Nineteen patients in 20 admission encounters met criteria for being at risk for having PARDS, and in all 20 patient encounters the patient’s parent consented to study participation. One patient (number 6) was excluded as the primary team caring for this patient believed that his multiple congenital anomalies contributed to his decompensation and intubation during an imaging study rather than an infection; however, six weeks later this patient (number 14) was readmitted and the parents again consented to the study. Table 1 details characteristics of the 19 enrolled patients.

**Table 1:** Patient Demographics and Clinical Characteristics.

Patient	Age	Sex	Race/Ethnicity^a^	Weight (kg)	PRISM/PELOD	Initial PARDS Stratification	Virus	Respiratory Culture	Ventilator-free Days^b^	Comments
1	9 d	Female	White	2.8	21/5	Mild	RSV^f^	No growth	24^d^	
2	1.5 y	Female	White	9.7	6/1	At risk	RSV^f^/Influenza	Not tested	28	Never intubated, blood sample only
3	3.3 y	Male	Black	21.2	22/4	Mild	Influenza A	MRSA	23^d^	
4	1.5 y	Male	White	8.7	6/5	Mild	Adenovirus	*Enterococcus* faecalis	9	Pseudohypoaldosteronism; HFOV^e^
5	1.6 y	Male	Black	18.4	27/5	Moderate	Influenza A	No growth	25	
7	6 m	Male	Black	6.6	9/3	At risk	Rhinovirus	*Haemophilus* influenza	21^d^	
8	1.4 m	Male	White	3.2	12/6	At risk	Rhinovirus	*Moraxella* b. catarrhalis	25	
9	1.2 y	Male	Black	9.7	8/4	At risk	Influenza A	No growth	21	Pleural effusion, chest tube
10	4.2 m	Male	Black	5.6	8/5	At risk	Rhinovirus	*Haemophilus* influenza	25	
11	1.5 m	Male	Black	5.5	17/7	At risk	RSV^f^	*Haemophilus* influenza	24	
12	1.7 y	Male	Black	13.5	28/7	At risk	Rhinovirus	*Staph* aureus	21	
13^b^	1.9 y	Female	Black	11.2	15/7	Severe	Coronavirus NL63	No growth	0	HFOV^e^, VA-ECMO^b^ Pulmonary hemorrhage
14^c^	5.5 m	Male	White	5.2	23/8	At risk	Rhinovirus	No growth	21^d^	Tracheomalacia, multiple congenital anomalies
15^b^	1.5 y	Female	Black	8.7	27/9	Severe	Parainfluenza 3	*Moraxella* catarrhalis	11	HFOV^e^, VA-ECMO^b^ Pulmonary hemorrhage
16	16 y	Female	White	68.5	27/9	Moderate	Influenza B	*Streptococcus* pyogenes	24	Pleural effusion, chest tube
17	1.9 y	Female	Biracial	11.8	12/6	Mild	Not performed	MRSA	24	
18	8.1 m	Female	White	8.2	26/9	Moderate	Not performed	Yeast	20	
19	1.2 m	Female	White	3.6	16/7	At risk	Negative	*Haemophilus* influenza	25	
20	10.6 y	Male	Black	23	18/7	Mild	Rhinovirus/HMPV^f^	Gm + cocci in pairs/Gm- rods & diplococci	23	

^a^None of the patients enrolled were of Hispanic ethnicity.

^b^Patients 13 and 15 were cannulated to venous-arterial extracorporeal membrane oxygenation.

^c^Patient 14 was enrolled into the study twice during unrelated admissions 6 weeks apart. There was no infection suspected during the first admission (enrolled as patient 6), therefore only data from the second admission is included.

^d^Patients 1, 3, 7, and 14 were reintubated within 48 hours of extubation.

^e^High frequency oscillatory ventilation.

^f^RSV: respiratory syncytial virus, HMPV: human metapneumovirus.

### Change in phenotype of neutrophils after airway migration from the circulation

**Figure 1:**
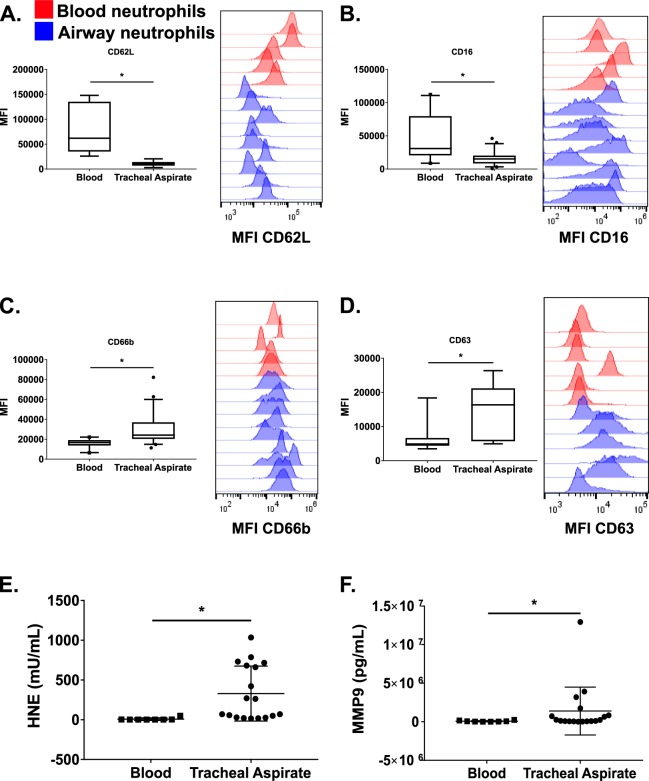
Characterization of blood and airway neutrophils markers by flow cytometry and markers of neutrophil activation in the plasma and airway fluid. Box plots of neutrophil cell surface markers for (**A**) CD62L, (**B)** CD16, (**C**) CD66b, and (**D)** CD63 in the blood (n = 8) and airway (n = 18) collected within 24 hours of endotracheal intubation (Day 1). Examples of histograms of the primary flow cytometry data are shown beside the box plot for each marker. Red and blue histograms represent blood and airway neutrophils, respectively. (**E**) Human neutrophil elastase activity assay and (**F**) matrix metalloproteinase 9 (MMP-9) protein levels from Day 1 plasma and cell-free airway fluid from tracheal aspirate samples. Box plots depict median values, the box edges are the 25^th^ to 75^th^ interquartile ranges (IQR), and the whiskers are the 5−95% confidence intervals. ^*^p < 0.05.

Using flow cytometry, we compared the expression of critical surface markers between blood and airway neutrophils in children with acute lower airway infections within 24 hours of endotracheal intubation. Representative flow cytometry histograms showing the heterogeneity of surface marker expression in airway and blood neutrophils are shown in Fig. 1A–D. Both L-selectin (CD62L) and CD16, the low affinity Fc gamma receptor for immunoglobulin G (IgG), which can mediate degranulation, phagocytosis, and oxidative burst, were shed upon airway migration. Cell surface expression of CD63, a marker of primary granule exocytosis, and CD66b, a marker of secondary granule exocytosis, were increased in the airway compared with blood neutrophils. Granule release by airway neutrophils was confirmed by the presence of significant activity of human neutrophil elastase (HNE), a protease contained within primary granules, and matrix metalloprotein 9 (MMP-9) protein levels, a protease contained within secretory granules, in the cell-free airway fluid compared with platelet-free plasma (Fig. 1E,F).

**Figure 2:**
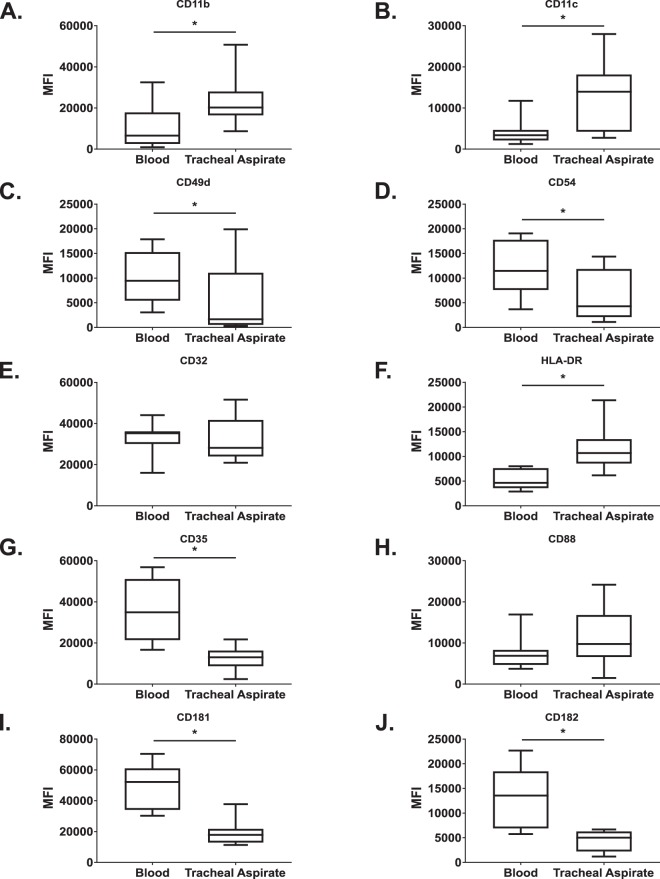
Characterization of blood (n = 8) and airway (n = 18) neutrophil cell surface markers of activation by flow cytometry. Samples were collected within 24 hours of endotracheal intubation (Day 1). Surface expression of integrins (**A**) CD11b, (**B**) CD11c, (**C**) CD49d, and (**D**) CD54, the immunoglobulin G receptor (**E**) CD32, (**F**) MHC II receptor, HLA-DR, and complement receptors (**G**) CD35 (C3b/C4b, CR1), and (**H**) CD88 (C5aR), and of the IL8 receptors (**I)** CD181 and (**J**) CD182. Box plots depict median values, the box edges are the 25^th^ to 75^th^ interquartile ranges (IQR), and the whiskers are the 5−95% confidence intervals. ^*^p < 0.05.

We further characterized differences in surface expression of integrins responsible for adhesion and activation of the neutrophil respiratory burst, complement/antigen presentation, and IL8 chemotaxis receptors in airway vs. blood neutrophils (Fig. 2). CD11b (integrin alpha M) and CD11c (integrin alpha X) were increased, while CD49d (VLA-4α) and CD54 (ICAM-1) were decreased on airway compared to blood neutrophils. The antigen presenting receptor HLA-DR (major histocompatibility class II) was also increased in airway compared with blood neutrophils. CD35 (complement receptor 1) was decreased in airway compared to blood neutrophils, while CD88 (complement receptor 5) and CD32 (medium affinity IgG receptor, which mediates phagocytosis, oxidative burst, platelet aggregation and immunomodulation) were unchanged. Finally, both CD181 (CXCR1 or IL8RA) and CD182 (CXCR2 or IL8RB) receptors were decreased in the airway neutrophils compared to blood neutrophils.

### Human neutrophil elastase activity is increased in the airway fluid of children with bacterial respiratory co-infections

**Figure 3:**
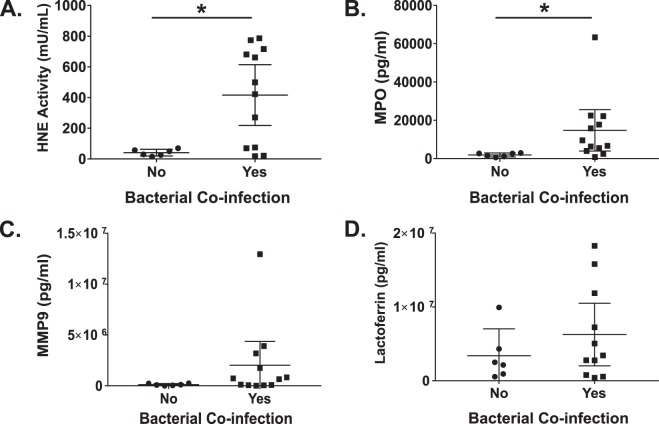
Airway supernatant (ASN) markers of neutrophils activation analyzed by the absence (n = 9–11) or presence (n = 19–21) of a respiratory bacterial co-infection from tracheal aspirate samples obtained within 24 hours of endotracheal intubation (Day 1 samples). (**A**) Human neutrophil elastase (HNE) activity and (**B)** myeloperoxidase levels are elevated in children with a respiratory bacterial co-infection compared to those without a bacterial co-infection. There is no significant difference in (**C**) matrix metalloproteinase 9 (MMP-9) or (**D**) lactoferrin protein levels in children with a respiratory bacterial co-infection compared to those without a bacterial co-infection. The central line is the mean value with whiskers representing the standard deviation. Samples were compared using the Mann-Whitney U test. ^*^p < 0.05.

Neutrophil degranulation is associated with increased severity of lung disease due to infection. HNE activity increased with versus without airway bacterial coinfection (Fig. 3). In addition to activity of HNE (Fig. 3A), MPO levels, but neither lactoferrin nor MMP-9, were higher in ASN from children with bacterial coinfection in the airways than those without (Fig. 3B–D).

### *In vitro* modeling of airway neutrophil conditioning in PARDS patients

**Figure 4:**
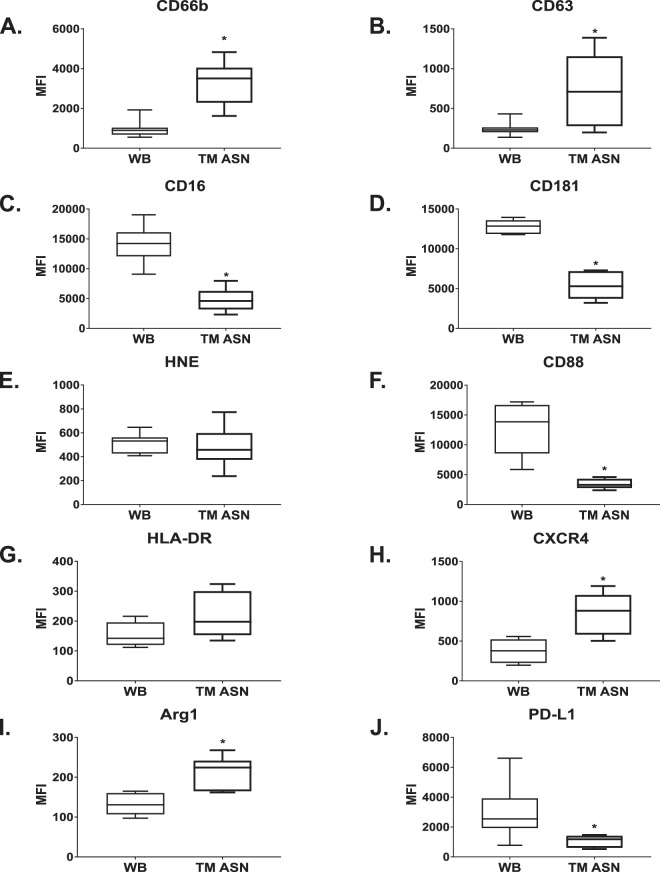
Characterization of cell surface markers of activation by flow cytometry of donor neutrophils from whole blood (WB) (n = 6) and transmigrated through the small-airways model for 14 hours towards airway supernatant (TM ASN) (n = 6–9). (**A**) CD66b, (**B**) CD63, (**C**) CD16, (**D**) CD181, (**E**) HNE, (**F**) CD88, (**G**) HLA-DR, (**H**) CXCR4 (CD184), (**I**) Arg-1, (**J**) PD-L1. Box plots depict median values, the box edges are the 25^th^ to 75^th^ interquartile ranges (IQR), and the whiskers are the 5−95% confidence intervals. Samples were compared using the Mann-Whitney U test. ^*^p < 0.05.

In order to further characterize differences in neutrophils recruited to the airways of children with or without bacterial coinfections following lower airway viral infections, we used ASN from children with lower airway viral infection collected within 24 hours of endotracheal intubation to show that this pathological milieu induces transepithelial migration of blood neutrophils in this *in vitro* model. Similar modulation of CD66b, CD63, CD16, and CD181 were observed between primary airway neutrophils from patients and donor neutrophils transmigrated into patient ASN compared to blood neutrophils (pre-transmigration) (Fig. 4A–D). Although patient ASN had intrinsic HNE activity, there was no evidence for surface recapture of HNE in transmigrated neutrophils (Fig. 4E). Unlike the *in vivo* patient data, CD88 (complement receptor 5a) was decreased in neutrophils transmigrated to ASN (Fig. 4F), and HLA-DR was not different in neutrophils transmigrated to ASN (Fig. 4G) compared with blood neutrophils. *In vivo*, CXCR4 is a surface marker expressed on human neutrophils that plays a role in tissue retention^25–27^. Upon neutrophil transmigration *in vitro*, CXCR4 was upregulated compared to blood neutrophils (Fig. 4H). Exploration of the Arg1 and PD-L1 axis demonstrated that Arg1 expression was increased on the cell surface of neutrophils transmigrated to ASN (Fig. 4I). Neutrophils transmigrated into pooled Day 1 ASN from all patients had decreased PD-L1 surface expression compared to blood neutrophils (Fig. 4J). However, we did not detect significant differences in Arg1 and PD-L1 expression on blood and airway neutrophils *in vivo* (data not shown). Experimental controls using negatively selected neutrophils rather than whole blood neutrophils (Fig. E3) and direct incubation of neutrophils with ASN compared with transmigration (Fig. E4) did not show significantly different results compared with those shown in Fig. 4, except for CD16 which decreased in the co-incubated experiment compared to transmigrated neutrophils.

### Respiratory burst in blood neutrophils *in vivo* and after transmigration into patient ASN *in vitro*

**Figure 5:**
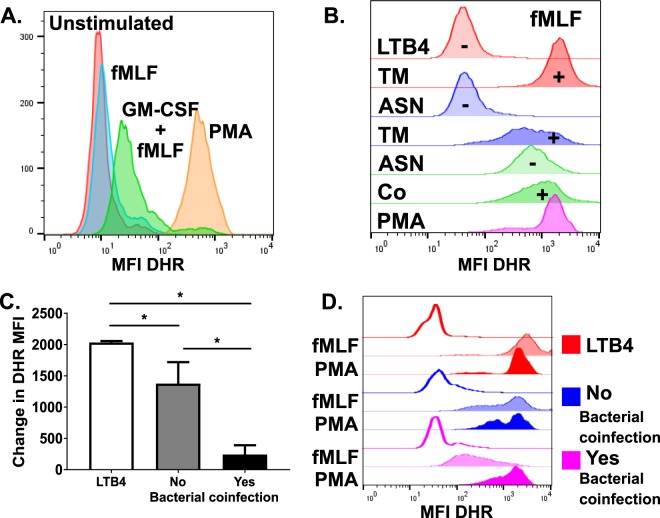
Transmigration through the airway model primes healthy donor neutrophils to activate the respiratory burst when stimulated by *N-*formylmethionyl-leucine-phenylalanine (fMLF) as measured by dihydrorhodamine (DHR) flow cytometry assay. (**A**) Representative flow cytometry histograms depicting healthy donor neutrophil DHR response to fMLF without (blue histogram) and with priming by GM-CSF (100 ng/mL; green histogram) for 30 minutes at 37 °C, 5% CO_2_. PMA-stimulated (100 pg/mL) neutrophils serve as a positive control (orange histogram). Unstimulated, DHR loaded neutrophils are shown as a negation control (red histogram). (**B**) Representative flow cytometry data depicting the DHR fluorescence to fMLF stimulus for transmigrated to LTB4 (red histograms), transmigrated to pooled Day 1 ASN (blue histograms) and neutrophils co-incubated with pooled Day 1 ASN (green histograms) for one donor. PMA (magenta histogram) is included as a maximal respiratory burst positive control. (**C**) Change in the DHR response to fMLF stimulation for neutrophils allowed to transmigrate for 14 hours toward LTB4, ASN with no bacterial coinfection (low HNE), or ASN with bacterial coinfection (high HNE) diluted 1:3 in serum-free media. Data are reported as the mean and standard deviation for three donors and analyzed using ANOVA with a *post-hoc* Tukey test for multiple comparisons. ^*^p < 0.05. (**D**) Representative flow cytometry histogram from one neutrophil donor transmigrated as described in C).

We next compared the respiratory burst capacity of blood neutrophils (Fig. 5A) to that of neutrophils transmigrated toward LTB4 (chemoattractant control) or patient ASN (Fig. 5B). For blood neutrophils, there was no response at rest or upon fMLF stimulus without initial priming with granulocyte macrophage-colony stimulating factor (GM-CSF), and the burst intensity upon GM-CSF + fMLF treatment was much lower than that seen with the PMA positive control (Fig. 5A). In contrast, neutrophils transmigrated toward LTB4 or pooled Day 1 ASN from all patients showed an increased respiratory burst intensity to fMLF compared to unstimulated transmigrated neutrophils (Fig. 5B). Coincubation of neutrophils with pooled Day 1 ASN, without transmigration, for the same duration in serum-free media resulted in stimulation of the respiratory burst with no increase in intensity with addition of fMLF. Neutrophils transmigrated toward ASN was bimodal with a diminished fMLF-stimulated respiratory burst compared with neutrophils transmigrated toward LTB4. Transmigration through the small airway epithelial model primes neutrophils for an increased respiratory burst to fMLF stimulation, similar in intensity to PMA stimulus, compared with blood neutrophils stimulated with GM-CSF (compare Fig. 5A,B).

Next, we compared neutrophils transmigrated to patient ASN with bacterial coinfection (corresponding to high HNE activity) to those transmigrated to patient ASN with no bacterial coinfection (corresponding to low HNE activity). As shown in Fig. 5C,D, neutrophils transmigrated to ASN from patients with bacterial coinfection had a lower fMLF-stimulated respiratory burst compared to those transmigrated to ASN from patients without bacterial coinfection. Neutrophils transmigrated to ASN from patients without bacterial coinfection had a lower respiratory burst than those transmigrated to LTB4. All conditions led to similar respiratory burst in response to PMA (solid histograms in Fig. 5D).

As seen in the healthy donor blood neutrophils, circulating neutrophils from patients also had low levels of priming as shown by a low respiratory burst with fMLF stimulation compared with PMA. The range of respiratory burst intensity at rest and after fMLF and PMA stimulation is illustrated in Fig. E5A–D, with some patients demonstrating blood neutrophils that are primed to respond to fMLF and some that are not (Fig. E5C,D).

Surface expression of CD16 and CD62L were measured on neutrophils from patient blood obtained within 24 hours of intubation, at rest and following stimulation with fMLF or PMA. Neutrophils that shed CD62L and have high CD16 expression have been previously associated with an immunosuppressive phenotype, while neutrophils with low levels of both CD62L and CD16 are considered immature^14,28–30^. We did not observe elevated percentages of either of these immunosuppressive or immature neutrophil populations in our patients on Days 1 and 3 (Fig. E5E). Furthermore, CD62L was lost from the cell surface when neutrophils were treated with fMLF or PMA (Fig. E5F).

### Altered surface protein expression and bacterial killing of neutrophils transmigrated to HNE-rich ASN

**Figure 6:**
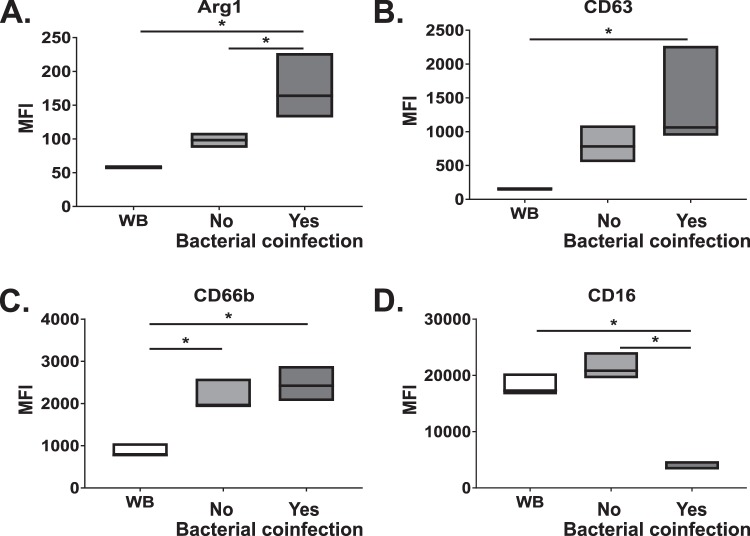
Box plots depict surface marker changes for neutrophils from 3 donors transmigrated as in C): (**A**) Arg1, (**B**) CD63, **C)** CD66b, and (**D**) CD16. Box plots depict median values, the box edges are the minimum and maximum values. ^*^p < 0.05.

We compared surface protein expression of markers in neutrophils transmigrated to HNE-rich and HNE-poor ASN. As shown in Fig. 6A–D, Arg1, CD63, CD66b were increased and CD16 expression was decreased in neutrophils transmigrated to bacterial coinfected patient ASN versus no bacterial coinfection patient ASN (Fig. 6A–D). The complete set of surface markers assessed in those two conditions, in comparison with blood neutrophils (pre-transmigration) and with neutrophils transmigrated to LTB4 is shown in Fig. E6.

**Figure 7:**
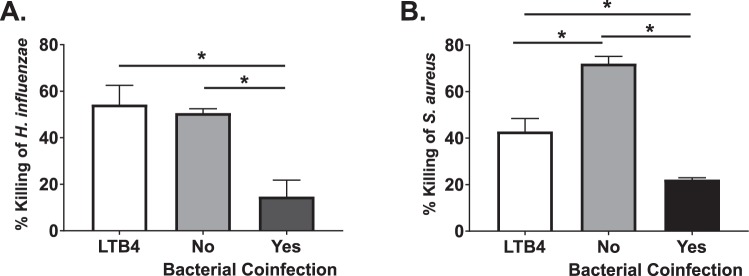
Percent bacterial killing of (**A**) *Haemophilus influenzae* or (**B**) *Staphylococcus aureus* by neutrophils from 3 donors allowed to transmigrate to LTB4, or ASN with no bacterial coinfection (low HNE) or ASN with bacterial coinfection (high HNE) for 14 hours. Samples were compared using ANOVA with a *post-hoc* Tukey test for multiple comparisons. ^*^p < 0.05.

Finally, we performed bacterial killing assays by neutrophils of *H*. *influenzae* and *S*. *aureus*, which are PARDS-associated gram-negative and gram-positive bacteria, respectively. Neutrophils transmigrated toward bacterial coinfected ASN showed a lower ability to kill both *H*. *influenzae* and *S*. *aureus* than those transmigrated to no bacterial coinfection ASN (Fig. 7). Neutrophils transmigrated toward LTB4 killed *H*. *influenzae* with similar efficiency as those transmigrated to HNE-poor ASN, while for *S*. *aureus* they were less efficient (Fig. 7).

## Discussion

Blood neutrophils recruited to the lungs of children at risk of or with PARDS due to lower airway viral infections are generally believed to rapidly die therein, leading to the passive release of HNE and other toxic by-products that promote lung injury^31–33^. In contrast with this conventional paradigm, the findings presented here support the notion that a large population of neutrophils remains viable and undergo profound changes upon recruitment to the airways of these patients. Viable airway neutrophils demonstrated surface mobilization of multiple sets of granules, and lose surface expression of CD16, a key phagocytic receptor. Furthermore, airway neutrophils increased expression of HLA-DR, typically associated with professional antigen-presenting cells, thus reflecting functional changes. To further investigate this process, we adapted an airway inflammation model developed in our group to study diseases of chronic lung inflammation (CF, asthma, COPD) to acute lung inflammatory processes occurring in children with acute respiratory failure due to lower airway infections. This model allowed for transepithelial recruitment and pathological conditioning of neutrophils by cell-free patient airway fluid and recapitulated cardinal features of airway neutrophils collected from these patients *in vivo*. In addition, we identified an association between active HNE exocytosis and bacterial coinfections within 24 hours of endotracheal intubation in these patients and observed that neutrophils recruited to the cell-free airway fluid of patients coinfected with bacteria become deficient in their respiratory burst capacity and in their ability to kill bacteria.

HNE is a serine protease contained in primary granules, and elevated levels in bronchoalveolar lavage and plasma are associated with severity of lung injury in ARDS^34^. While extracellular HNE can reflect live neutrophil activation and degranulation, in the tracheal aspirate both necrotic and neutrophils releasing neutrophil extracellular traps (NETs) may also contribute to increased extracellular levels of HNE, thus precluding the use of extracellular HNE activity as a stringent marker of live neutrophil degranulation. Elevated levels of MMP-9 in the lung epithelial lining fluid of patients with ARDS has also been associated with increased severity of lung injury^34,35^. Primary granule exocytosis in airway compared to blood neutrophils is evidenced by the increase in CD63 expression in addition to decreases in CD16 and CD35 expression as the latter proteins contain HNE and MMP cleavage sites and may also be removed by membrane reuptake^9,36^. While CD62L downregulation is expected, CD16 expression is generally increased upon transmigration due to degranulation of secretory vesicles and tertiary granules^37,38^; however, CD16 can be downregulated if endocytosed and cleaved by high activity levels of HNE and MMP-9^16,36^. Secondary granule exocytosis in airway neutrophils is evidenced by an increase in CD66b expression compared to blood neutrophils. Whether this abnormal activation response can be leveraged therapeutically remains unclear. Indeed, HNE inhibitors, such as sivelestat, have failed to improve mortality in ARDS patients likely due to competing proteases, release of other damage-associated molecular pattern molecules and alarmins, and the immunomodulatory effects of neutrophils on T-cells in the airways of ARDS patients^39^. Significant differences in neutrophil activation markers between the blood and airway compartments observed in our patient population is consistent with prior studies^5,9,16,22,40^. Our results highlight the importance of studying the airway fluid and cells recruited therein, rather than simply inferring airway neutrophil behavior and function based on cells isolated from blood.

Neutrophil priming by agents such as GM-CSF, tumor necrosis factor α or lipopolysaccharide delay apoptosis of neutrophils at sites of inflammation and increase their degranulation and respiratory burst responses to an activating agent^5,41^. Interestingly, one of the roles assigned to the healthy pulmonary vasculature is to selectively retain primed neutrophils, deprime and release them into the bloodstream^42^. This depriming mechanism may be dysfunctional in ARDS, resulting in release of primed neutrophils into the systemic vasculature that can damage extra-pulmonary organs^42^. Migration of healthy donor neutrophils through our small airway epithelial model primes these neutrophils to undergo a respiratory burst; however, migration to ASN with high NE activity had an attenuated respiratory burst upon fMLF stimulation. The decreased respiratory burst in the presence of HNE-rich ASN from bacterially coinfected patients may serve as rapidly assessed biomarker of bacterial coinfection. The mechanism to explain the poor killing of *H*. *influenzae* and *S*. *aureus* by neutrophils transmigrated to HNE-rich ASN in our model, which is associated with the clinical outcome of a bacterial coinfection, remains to be explored. An increase in relative Arg1 gene transcript levels has been shown in blood neutrophils from ARDS patients relative to healthy volunteers^5^ consistent with our finding of an upregulation in Arg1 surface expression in neutrophils transmigrated to bacterial coinfected ASN. The influence of other immunoregulatory pathways, such as the PD-L1/PD-1 axis, are under investigation as a possible mechanism to explain bacterial coinfection in some children for viral induced respiratory failure.

While our transepithelial neutrophil migration model sets up the possibility for controlled studies of the fate of neutrophils in PARDS patients, we acknowledge several limitations. First, we did not perform full functional analyses on primary airway neutrophils due to limited cellular material from patients. Instead, we used cell surface markers of activation as a proxy to inform how well our transepithelial migration model compared to the changes seen in blood and airway neutrophils from patients. In addition, most of our experimental data comes from the transepithelial migration model which recapitulates the small airways – respiratory generation 8 to 16. The tracheal aspirates from the patients were actually collected from airway generation 1 to 2; however, bronchoalveolar lavage of children is not commonly performed unless needed for diagnostic and therapeutic purposes for severe lung injury. Second, we are limited in our ability to collect blood samples from children with a weight under 6 kg or without a central venous or arterial catheter for blood sampling, which made paired comparisons between blood and airway neutrophils from each patient impossible. Nevertheless, despite our limited numbers of patients, we sampled enough patients to identify significant differences in activation and immunomodulatory markers on neutrophils. Although we previously showed differential neutrophil behavior comparing hypertonic saline-induced sputum cells from CF patients and healthy adults^9,16,22^, we were not able to include controls in this study owing to ethical limitations on obtaining non-clinically indicated airway lavage samples on children intubated for elective surgeries. In addition, children intubated for airway protection, rather than for respiratory failure due to a pulmonary cause, do not have neutrophil-rich airway lavage samples making a direct comparison of cellular material impossible to perform. Instead, we focused on serial blood and airway compartment sampling over time in the PICU to profile how neutrophils change such that each patient may act as their own control in future studies. While children in this study ranged in age from 9 days to 16 years, the majority were under 2 years of age limiting comparisons of neutrophil behavior stratified by age. Finally, patients included in this study were infected with a variety of respiratory viruses and due to limited sample size, we cannot determine the effect of any one virus on airway neutrophil behavior or susceptibility to bacterial coinfection. Despite the infectious heterogeneity of our limited number of samples, we were able to detect a significant neutrophil dysfunction signal in the presence of an activated neutrophil environment.

In summary, our study highlights phenotypic differences between airway and blood neutrophils in patients at risk of or with PARDS due to lower respiratory tract viral infections with or without bacterial coinfection, emphasizing the need to study compartment-specific behaviors of neutrophils. In addition, we showed that bacterial coinfections on Day 1 of illness may be predicted using markers of neutrophil granule release, immunomodulatory proteins, and metabolic activities, which may inform antimicrobial treatment of patients. Finally, we showcased a novel *in vitro* transmigration model to study airway neutrophil function following recruitment to and conditioning by patient cell-free airway fluid, laying critical foundations for future mechanistic and therapeutic studies of airway neutrophils in PARDS.

## Supplementary information

Supplementary Data

## Data Availability

The datasets generated during and/or analyzed during the current study are available from the corresponding author on reasonable request.
