# Supplementary material for: Neutrophil Dysfunction in the Airways of Children with Acute Respiratory Failure Due to Lower Respiratory Tract Viral and Bacterial Coinfections
Source: Sci Rep. 2019 Feb 27;9:2874. doi: 10.1038/s41598-019-39726-w (PMC6393569; doi:10.1038/s41598-019-39726-w)
Supplement: Supplementary file 1 — Supplementary Data [file 41598_2019_39726_MOESM1_ESM.docx]

**NEUTROPHIL DYSFUNCTION IN THE AIRWAYS OF CHILDREN WITH ACUTE RESPIRATORY FAILURE DUE TO LOWER RESPIRATORY TRACT VIRAL AND BACTERIAL COINFECTIONS**

Jocelyn R. Grunwell, Vincent D. Giacalone, Susan Stephenson, Camilla Margaroli, Brian S. Dobosh, Milton R. Brown, Anne M. Fitzpatrick, Rabindra Tirouvanziam

**Online Data Supplement**

**Table E1. Human Antibodies used for Flow Cytometry**

| **Antibody** | **Clone** | **Fluorophore** | **Supplier** | **Panel** |
| --- | --- | --- | --- | --- |
| CD62L | DREG-56 | BV421 | BioLegend | N1/Transmigration |
| CD41a | HIP8 | Pacific Orange | BioLegend | All |
| CD11b | M1/70 | AF488 | BioLegend | N1 |
| CD66b | G10F5 | PerCPCy5.5 | BioLegend | All |
| Arg1 | 14D2C43 | PE | BioLegend | N1/Transmigration |
| CD11c | 3.9 | PECy7 | BioLegend | N1 |
| PD-L1 | 29E.2A3 | APC or PECy7 | BioLegend | N1/Transmigration |
| CD16 | 3G8 | APCCy7 | BioLegend | All |
| CD63 | H5C6 | Pacific Blue | BioLegend | N2 |
| CD32 | FUN-2 | FITC | BioLegend | N2 |
| CD35 | E11 | PE | BioLegend | N2 |
| CD88 | S5/1 | PECy7 | BioLegend | N2 |
| CD49d | 9F10 | APC | BioLegend | N2 |
| HLA-DR | L243 | BV421 or FITC | BioLegend | N3/Transmigration |
| CD181 | 8F1/CXCR1 | AF488 | BioLegend | N3 |
| CD86 | IT2.2 | PE | BioLegend | N3 |
| CD182 | 5E8/CXCR2 | PECy7 | BioLegend | N3 |
| CD54 (ICAM1) | HA58 | AF647 | BioLegend | N3 |
| CD56 | HCD56 | BV421 | BioLegend | Airway purity |
| CD14 | HCD14 | AF488 | BioLegend | Airway purity |
| CD3 | HIT3a | PE | BioLegend | Airway purity |
| CD15 | W6D3 | PECy7 | BioLegend | Airway purity |
| CD45 | HI30 | AF647 | BioLegend | Airway purity |
| HNE | 950317 | AF647 | R&D Systems | Transmigration |
| CD184 (CXCR4) | 12G5 | PE | BioLegend | Transmigration |

**Supplementary Figure Legends**

**Figure E1.** Flow cytometry gating strategy of neutrophils from **A**) whole blood and **B**) tracheal aspirate samples. Single cells were selected by forward scatter (FSC) – area versus FSC-height. Neutrophils were selected by gating on FSC-area versus side-scatter-area (SSC-A). Platelets, platelet-cell aggregates, and dead cells were excluded by gating on Live/Dead Aqua negative and CD41a^-^ versus SSC-A. Single, live neutrophils were confirmed by selecting for CD66b^+^/SSC-A^High^ cells.

**Figure E2.** Fluorescence minus (FM) controls of patient blood and airway neutrophils selected based on the gating strategy described and confirmed as being CD66b^+^/SSC-A^High^/CD16^+^. Mean fluorescence intensities (MFI) of the five remaining unstained fluorophore channels used in the flow panels are shown for: **A)** Pacific Blue (PB450), **B)** Alexa Fluor 488 (AF488), **C)** phycoerythrin-cyanine 7 (PECy7), **D)** phycoerythrin (PE), and **E)** allophycocyanin (APC). Box plots depict median values, the box edges are the 25^th^ to 75^th^ interquartile ranges (IQR), and the whiskers are the 5% - 95% confidence intervals and are intentionally scaled to correspond to the patient data shown in Figure 1 and 2.

**Figure E3.** Characterization of cell surface markers of activation by flow cytometry of neutrophils purified by negative selection prior to transmigration (n = 6) and donor neutrophils transmigrated through the small-airways model toward airway supernatant (TM ASN) for 14 hours (n = 6). **A)** CD66b, **B)** CD63, **C)** CD16, **D)** HNE, **E)** CD88, **F)** CD181, **G)** CXCR4 (CD184), **H)** HLA-DR, **I)** Arg-1, **J)** PD-L1. Box plots depict median values, the box edges are the 25^th^ to 75^th^ interquartile ranges (IQR), and the whiskers are the 5% - 95% confidence intervals. Samples were compared using the Mann-Whitney U test. **p* < 0.05.

**Figure E4.** Characterization of cell surface markers of activation by flow cytometry of neutrophils incubated in ASN (n = 3-6) and donor neutrophils transmigrated through the small-airways model for 14 hours towards airway supernatant (TM ASN) (n = 6-9). **A)** CD66b, **B)** CD63, **C)** CD16, **D)** HNE, **E)** CD88, **F)** CD181, **G)** CXCR4 (CD184), **H)** HLA-DR, **I)** Arg-1, **J)** PD-L1. Box plots depict median values, the box edges are the 25^th^ to 75^th^ interquartile ranges (IQR), and the whiskers are the 5% - 95% confidence intervals. Samples were compared using the Mann-Whitney U test. **p* < 0.05.

**Figure E5.** Analysis of cell surface markers, CD62L and CD16, and fMLF and PMA stimulated DHR respiratory burst on neutrophils from whole blood collected on Day 1 and Day 3 following endotracheal intubation from children with acute respiratory failure due to lower respiratory tract infections (n = 8 for each day). **A**) Blood neutrophils from patients are minimally stimulated by fMLF using the DHR respiratory burst assay. **B**) There is a robust, but variable DHR respiratory burst from patient blood neutrophils stimulated using PMA. **C & D**) Flow data from two different patients showing differential the DHR respiratory burst in response to fMLF (blue histograms) and PMA (orange histograms). Neutrophils from the patient data in panel **D**) are primed as demonstrated by an increase in DHR fluorescence to fMLF stimulus as compared to the neutrophils from a different in panel **C**) that are not primed as shown by a lack of DHR fluorescence in response to fMLF. **E**) The majority of neutrophils are CD62L^High^/CD16^High^ (mature), followed by CD62L^Low^/CD16^High^ (immunosuppressive), CD62L^High^/CD16^Low^ (immature), and CD62L^Low^/CD16^Low^ (progenitor) neutrophils (1, 2). DHR fluorescence data was analyzed using a paired t-test. **F**) Dot blot of representative flow cytometry data showing the relatively consistent expression of CD16 and the marked decrease in CD62L expression in response to fMLF (blue dots) and PMA (orange dots) stimuli compared to baseline, unstimulated expression (red dots).

**Figure E6.** Characterization of cell surface markers of activation by flow cytometry of neutrophils in whole blood from 3 donors and the corresponding negatively selected neutrophils transmigrated to LTB4, pooled ASN from 6 patients with low neutrophil elastase (NE) activity, or pooled ASN from 6 patients with high NE activity for 14 hours. **A)** CD66b, **B)** CD63, **C)** CD16, **D)** HNE, **E)** HLA-DR, **F)** PD-L1, **G)** Arg-1. Box plots depict median values and box edges are the minimum and maximum values. Samples were compared using ANOVA with a *post-hoc* Tukey test. **p* < 0.05.


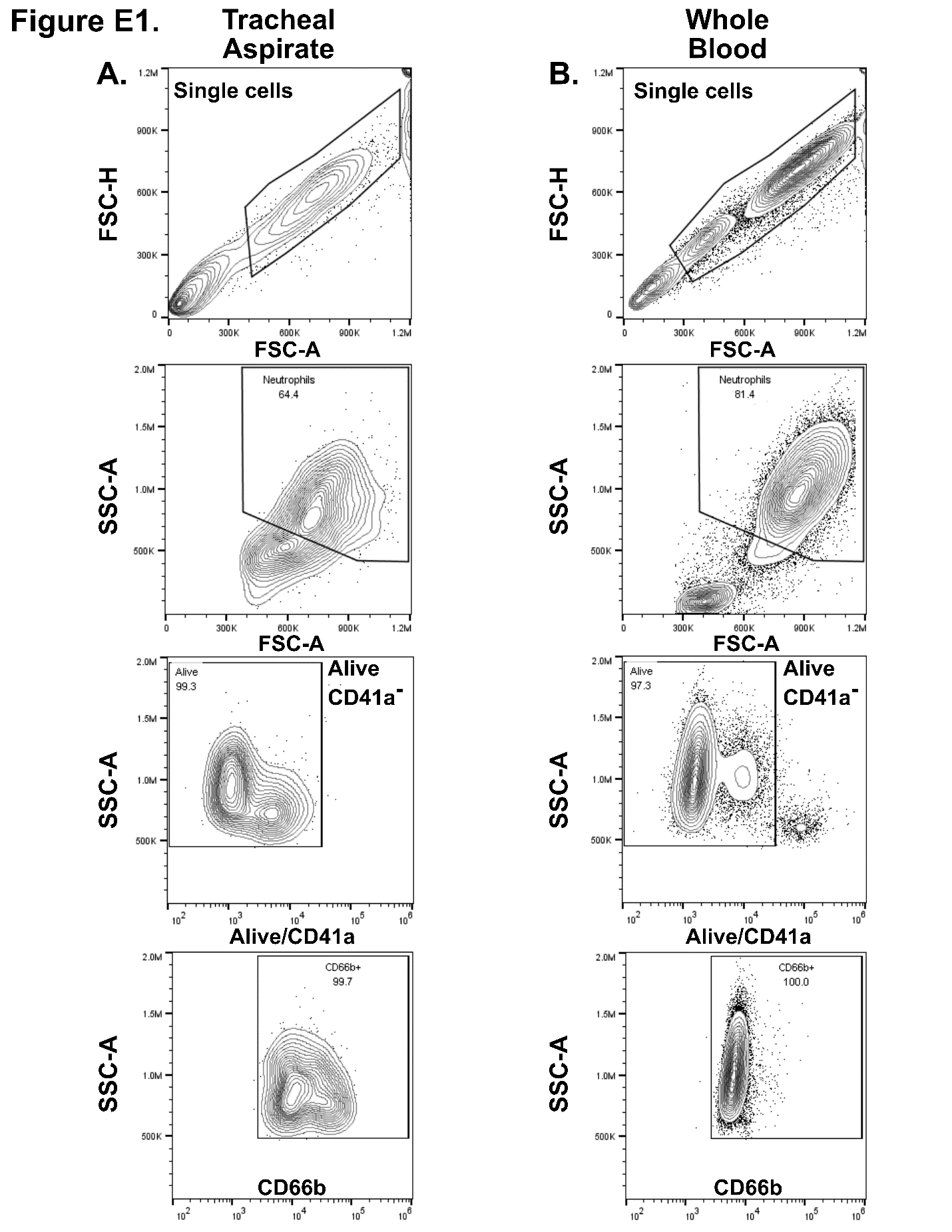


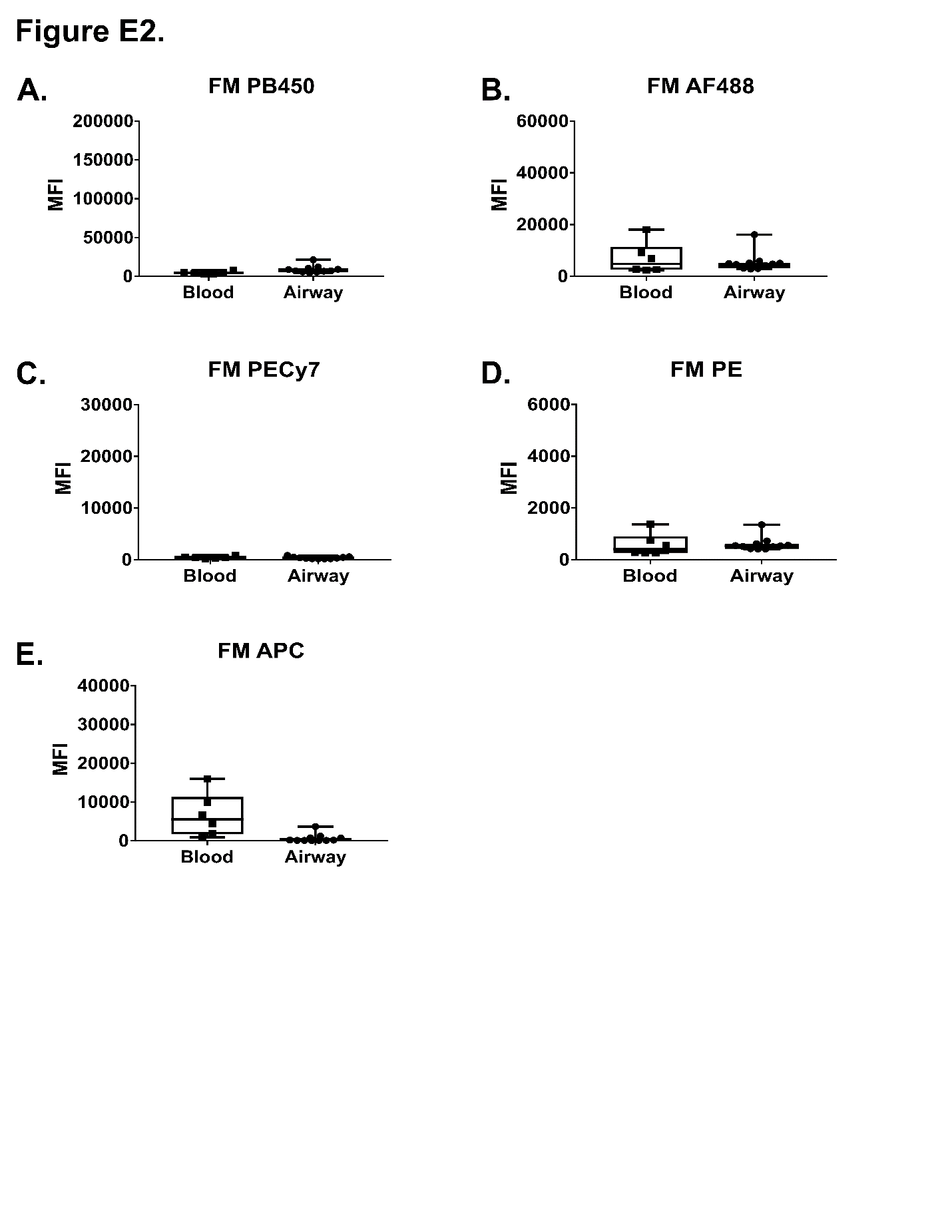


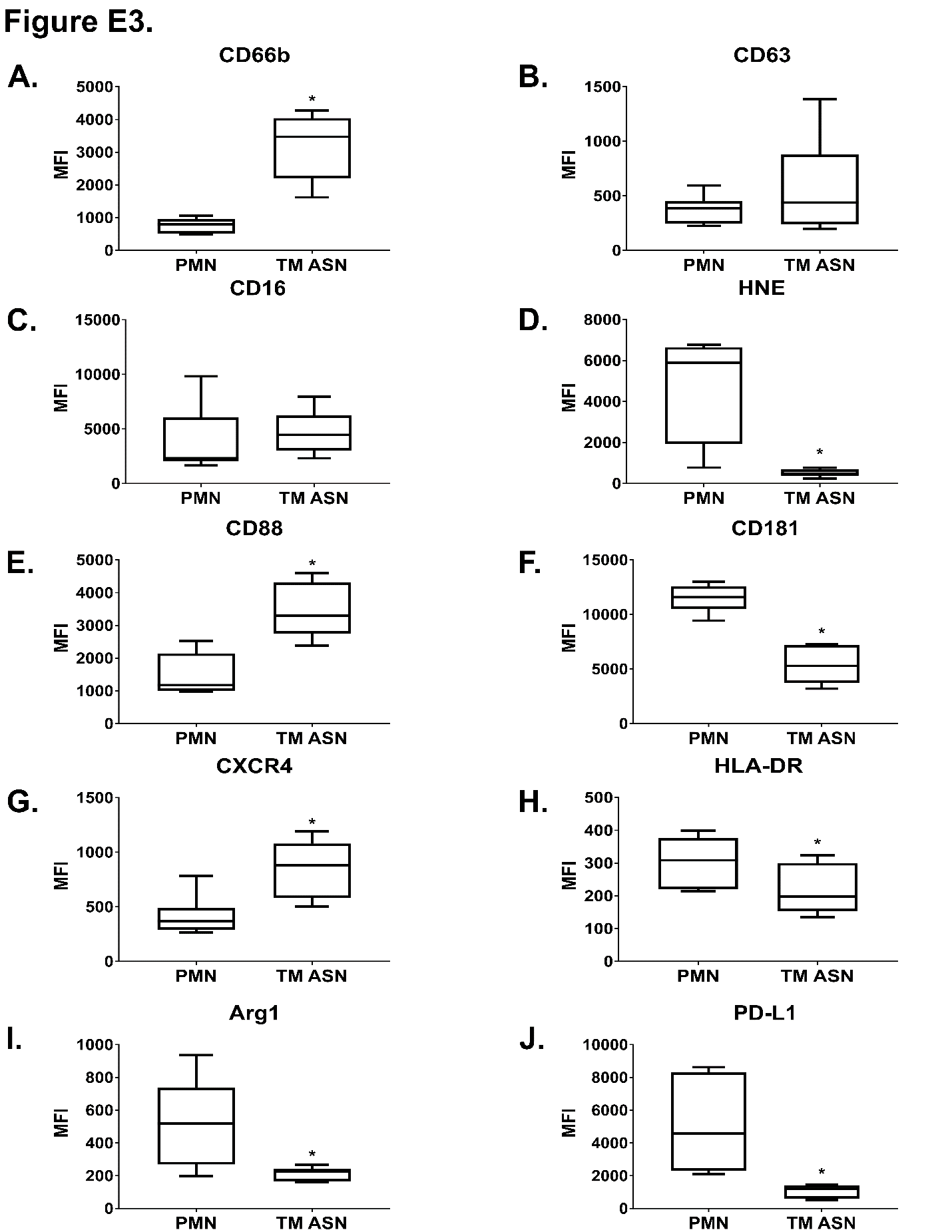


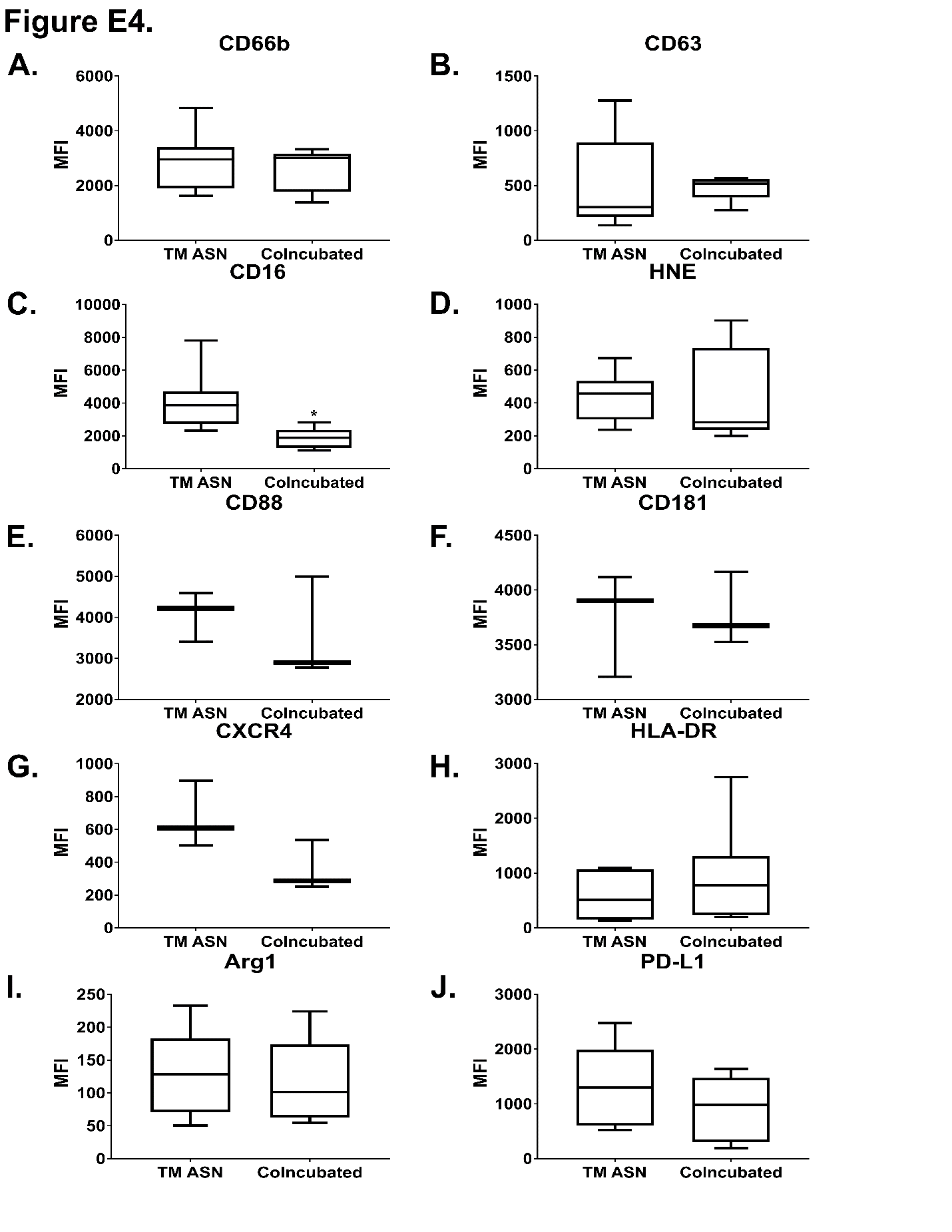


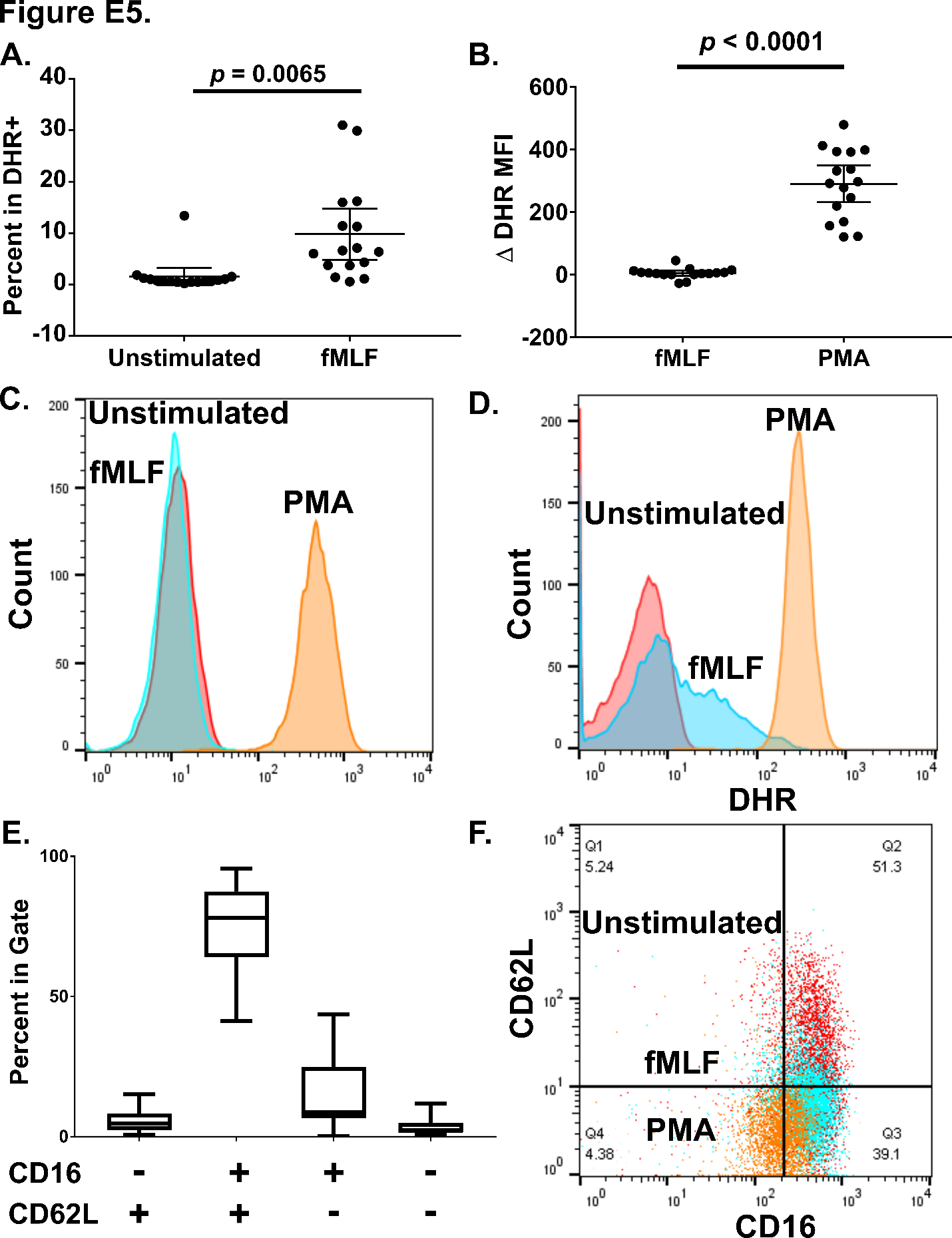


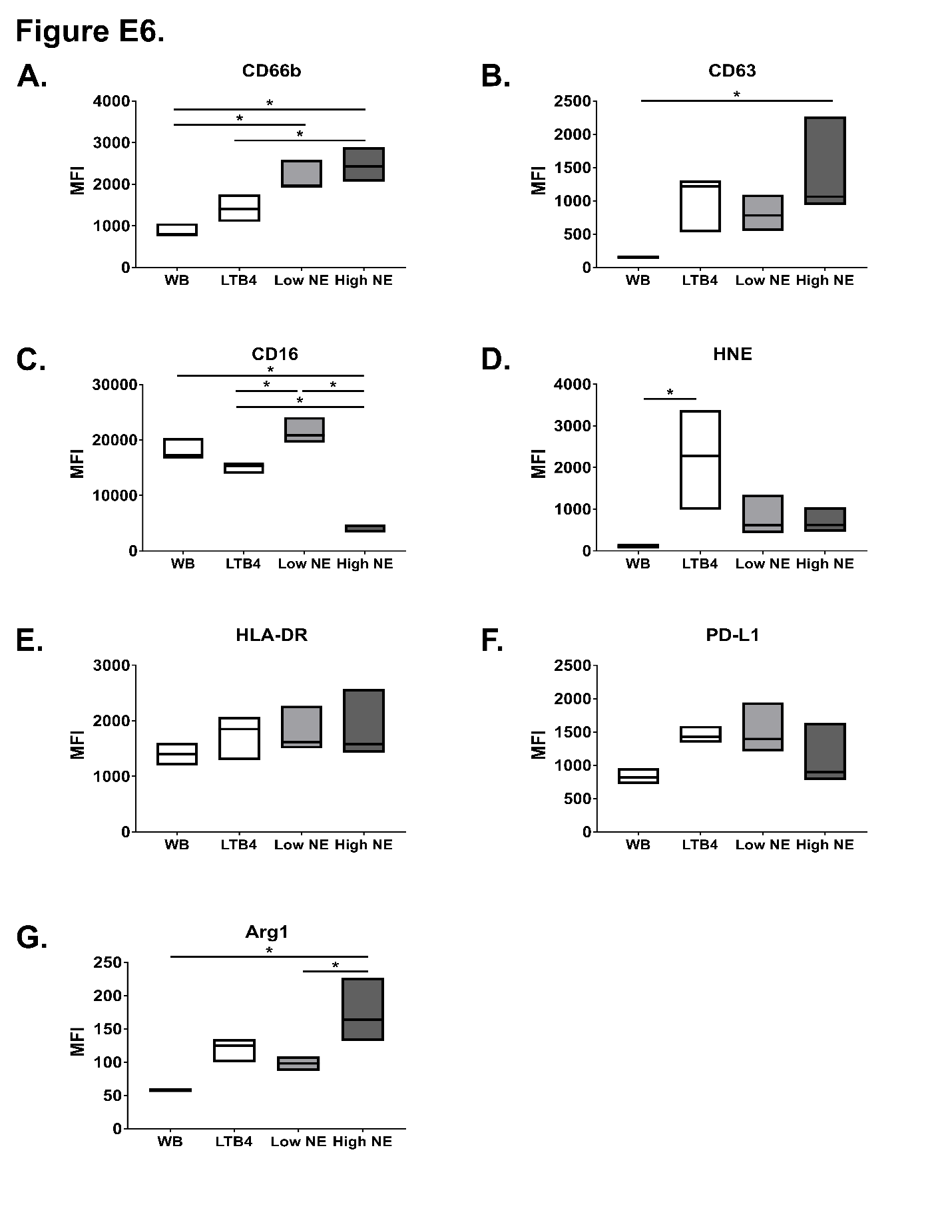


1. Pillay J, Kamp VM, van Hoffen E, Visser T, Tak T, Lammers JW, Ulfman LH, Leenen LP, Pickkers P, Koenderman L. A subset of neutrophils in human systemic inflammation inhibits t cell responses through mac-1. *J Clin Invest* 2012;122(1):327-336.

2. Pillay J, Ramakers BP, Kamp VM, Loi AL, Lam SW, Hietbrink F, Leenen LP, Tool AT, Pickkers P, Koenderman L. Functional heterogeneity and differential priming of circulating neutrophils in human experimental endotoxemia. *J Leukoc Biol* 2010;88(1):211-220.
